# Academic Half-Day Education Experience in Post-graduate Medical Training: A Scoping Review of Characteristics and Learner Outcomes

**DOI:** 10.3389/fmed.2022.835045

**Published:** 2022-03-02

**Authors:** Myong Sun Choe, Lynne C. Huffman, Heidi M. Feldman, Lauren M. Hubner

**Affiliations:** Division of Developmental-Behavioral Pediatrics, Department of Pediatrics, Stanford University School of Medicine, California, CA, United States

**Keywords:** medical education, academic half-day, post-graduate medical education, Kirkpatrick model, scoping review

## Abstract

**Background:**

The academic half-day (AHD) has grown in popularity for medical education because it intends to provide learners with uninterrupted, immersive learning time that may promote participant attendance, engagement, and knowledge. Little is known about the extent of use, forms, or effectiveness of AHD in Post-graduate medical education. This scoping review summarizes existing literature and describes the learning outcomes, according to the Kirkpatrick model of learning evaluation, of AHD experiences on Post-graduate medical trainees.

**Methods:**

Authors used Arksey and O'Malley's methodological framework, searching electronic scientific literature databases from the years of 1977–2019 with relevant key terms and identifying 735 papers. Two independent raters completed title/abstract screening and then extracted pertinent data from papers meeting specified criteria.

**Results:**

Authors identified 38 relevant papers published in English, originating from programs in US (*n* = 19) and Canada (*n* = 19), spanning 4 disciplines: Medicine (*n* = 17, 45%), Pediatrics (*n* = 10, 26%), Critical Care/Surgery (*n* = 9, 24%), Radiology (*n* = 2, 5%). A majority (*n* = 33, 87%) described specific educational experiences; most focused on residents only (*n* = 27). The educational experiences included various teaching strategies; few were didactics only (*n* = 4) and most were multi-modal including simulation, case-based learning, problem-based learning, and/or self-directed online study. AHD size ranged from 5 to 364 participants (median 39). AHD length was 1.5–6 h (median 3). Required resources were inconsistently described. When evaluations of the specific educational experience were reported (*n* = 35 studies), the majority of studies used weak research designs (e.g., one group, pre/post-test, *n* = 19); few studies used strong research designs (e.g., randomized controlled trial, *n* = 2). Positive effects of AHD ranged across Kirkpatrick levels 1–3 learner outcomes.

**Conclusions:**

The composition and content of AHD in Post-graduate medical education vary. Few studies of AHD use stringent research designs, and none include learner outcome measures at the highest Kirkpatrick level (i.e., level 4 results/patient outcomes). A consensus definition and further high-quality research on AHD in Post-graduate medical education is needed.

## Introduction

The Accreditation Council for Graduate Medical Education (ACGME) requires medical residency and fellowship programs to include a formal curriculum containing didactic sessions (1, 2). The programs must have educational sessions that include independent and group learning experiences to ensure that trainees attain the knowledge, skills, and attitudes necessary for competency. The ACGME suggests content areas of study but does not dictate specific teaching strategies or suggest preferred modality, frequency, format, or length.

Traditionally, Post-graduate medical training programs have fulfilled this requirement by providing daily noon conferences (NC) and/or other 1-h teaching sessions occurring several days per week throughout the training program (3). Cognitive science has consistently demonstrated that distributed practice is more effective than consolidated or massed practice for memory and skill development (4–6). However, many of such studies have measured the effects of the gap between exposures on later memory; the definitions of distributed practice used by psychologists include learning experience in spaced time with repeated information (7–9). Daily noon conference typically covers different learning topics each day, as opposed to strategic repetition of information, making it harder to directly apply these reported benefits of distributed practice.

Post-graduate medical training programs have long sought to provide feasible and effective teaching for their trainees, while also considering the demands of clinical service, duty hour restrictions, and limited resources. The evidence for the efficacy of traditional 1-h didactics (spaced/distributed learning) is conflicting (10). In recent years, trainees are frequently physically dispersed to different service sites; this feature makes it difficult to bring large numbers of trainees together for a short mid-day conference in a specific location. Resident learning is also chronically interrupted by frequent notifications and questions from inpatient wards (11, 12).

An alternative teaching strategy, casually known as the academic half-day (AHD), has grown in popularity because it provides a block of protected learning time that aims to improve trainees' attendance and active engagement without interruption (10). The assumption is that learning and knowledge acquisition is facilitated by uninterrupted massed practice over frequently disrupted distributed practice. A key feature of the AHD is a protected extended block of time for education; the precise length of time may not be specified. Little is known about the extent of application of the term, AHD teaching model (e.g., how often, duration, in what contexts, to what audience), the rationale for application of an AHD, the content of the AHD (e.g., range of topics, range of teaching modalities), or the effectiveness of the AHD as a teaching method in Post-graduate medical education (13). In this scoping review, we aim to identify and describe the existing literature on the implementation of the AHD as a teaching model in Post-graduate medical education curriculum, and explore range of learner outcomes described.

## Methods

We conducted a scoping review of the extant literature of AHD to map available literature and to highlight knowledge gaps about this educational strategy in Post-graduate medical education. Scoping reviews are commonly used as a tool to synthesize emerging research evidence (14, 15). According to Peters and colleagues, scoping reviews are useful when attempting to (1) map existing literature in terms of attributes and quantity; (2) clarify working definitions and conceptual boundaries of a topic or field; and, (3) identify gaps in existing literature and research (15). Scoping reviews are useful when considering a research area that has emerging evidence but a limited number of randomized controlled trials, making a systematic review unfeasible (14, 15). Our goal was to describe the characteristics of AHD as a teaching method in Post-graduate medical education, including the extent of use, content, and range of learner outcomes applying the Kirkpatrick model of learning evaluation (16). Kirkpatrick Model is an organizational tool that determines the effectiveness of educational interventions, commonly using a four-level framework to conceptualize learning outcomes.

The Arksey and O'Malley methodological framework for scoping reviews ensures an orderly approach to mapping the existing evidence on what is known broadly about an area of interest (17). We employed the five stages of the Arksey and O'Malley framework to describe the use of the AHD in Post-graduate medical training, and to identify any potential gaps in knowledge. These stages include: (1) identifying the research question; (2) identifying relevant studies; (3) selecting the studies; (4) charting the data; and (5) collating, summarizing, and reporting the results.

### Stage 1: Identifying the Research Question

This scoping review asked two research questions:

(1) What are the key characteristics of the AHD teaching method used in Post-graduate medical education curricula?
(2) What is known about effects of the AHD on Post-graduate medical trainees (i.e., learner outcomes)?

### Stage 2: Identifying Relevant Studies

We consulted with an experienced medical librarian to establish key information sources and identify available studies. We selected relevant electronic databases (PubMed, Web of Science, ERIC, MedEdPORTAL) to be searched from their respective inceptions to September 13, 2019. We also selected Google and Google Scholar to take advantage of the search engines' full-text search capability, something not available in some bibliographic databases. We then identified appropriate search terms: academic half-day, noon conference, didactic session, medical education, internship, residency, fellowship, and graduate. We identified the initial study count of 735 references that were imported into Covidence, a primary screening and data extraction tool (18). See Supplementary Data Sheet 1 for electronic database search strategies.

### Stage 3: Selecting Studies

**Figure 1:**
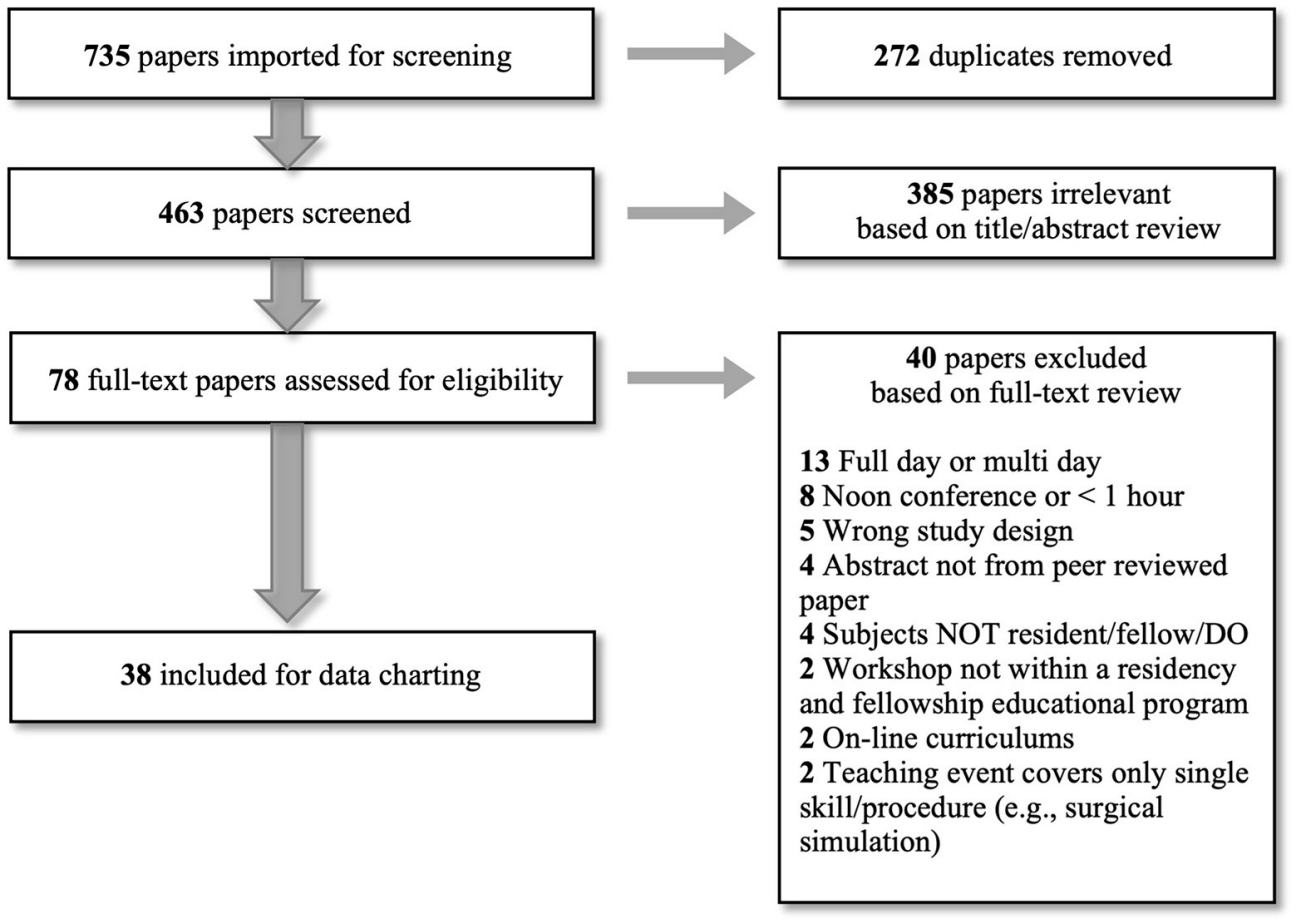
“Preferred Reporting Items for Systematic Reviews and Meta-Analyses” (PRISMA) flow chart.

Among those initially imported 735 references, we excluded 272 duplicated studies. Next, we established eligibility criteria for study selection to identify all studies describing the AHD as a teaching method in Post-graduate medical education. Inclusion criteria were: (1) Teaching was delivered to, or described for implementation with, Post-graduate medical trainees (MD/DO/residents/fellows), (2) Teaching was integrated into the general trainee curriculum, and (3) Teaching was described as a single intensive learning event (i.e., more than a single-hour didactic lecture), or self-described as a paper about AHD. Exclusion criteria were: (1) Papers written in a language other than English, (2) Teaching was provided during a workshop or other training opportunity not within the general residency or fellowship educational training program, (3) On-line curriculum solely, (4) Abstract not from peer-reviewed paper, (5) Review papers, (6) Teaching event covered only a single skill or procedure (e.g., surgical simulation of a single skill), and (7) Full day or multi-day lectures/trainings. Using these defined eligibility criteria, two authors (MSC and LMH) completed initial screening of the remaining 463 papers using the title and abstract. After title and abstract review, we excluded 385 papers. We then assessed 78 papers for full-text eligibility, at which point, we excluded 40 papers which resulted in 38 papers eligible for data charting and analysis. Through discussion with a third author (LCH), we resolved disagreements about study inclusion or exclusion at each step. See Figure 1 for summary.

### Stage 4: Charting Data

We employed Arksey and O'Malley's “descriptive-analysis” method of data extraction, summarizing information from the selected studies. Two authors (MSC and LMH) collectively developed a data-charting form using Microsoft^®^ Excel for Mac (version 16.41, 2020) to create a common framework. Each author then recorded the data independently on the data-charting form. The data-charting form included demographic data (e.g., title of article, name of author, year of publication, name of journal, country of publication, discipline), teaching characteristics (e.g., self-description as AHD, rationale, structure, teaching modality, delivery time), resources (e.g., number of faculty, space, money, teaching equipment, use of standardized patient), learner characteristics (e.g., target audience, number of participants), and study characteristics (e.g., evaluation design, method). The form also included information about any measurement of AHD effect (e.g., learner outcomes); this information was organized using the Kirkpatrick Model of Training Evaluation (16), as a framework for relating learner outcomes. Kirkpatrick model is classified into four levels: reaction, learning, behavior, and results. As applied to medical education evaluation, Level 1, reaction, assesses learner's reaction to the educational experience (e.g., learner attendance, interruption, or satisfaction). Level 2, learning, explores the degree of actual acquirement in intended knowledge, skill, and/or attitude, based on the participation in the educational experience. Level 3, behavior, looks into the degree of application of the educational experience to clinical practice on the job. Level 4, results, measures the effect of the educational experience on targeted goals such as patient outcome. See Supplementary Data Sheet 2 for a selective summary of information collected with this data charting form.

### Stage 5: Reporting Results

After independent completion of the data-charting form, we aggregated the information in a master Excel spreadsheet. All data disagreements were noted. The two authors (MSC and LMH) then collaboratively reviewed the aggregated data in the master spreadsheet and reconciled data differences, returning to the study in question if needed to re-assess, with input from third author (LCH) to resolve any disagreements.

## Results

We identified 38 papers (Figure 1) eligible for data charting and analysis published between 1977 and 2019. Over half of those papers were published in 2014 or later. Twenty-three papers self-identified a specific focus on AHD. The papers represented programs in the US (*n* = 19, 50%) and Canada (*n* = 19, 50%). The papers spanned 4 main disciplines: Medicine (*n* = 17, 45%), Pediatrics (*n* = 10, 26%), Critical Care/Surgery (*n* = 9, 24%), Radiology (*n* = 2, 5%). Medicine included Geriatrics, Family Medicine, Hematology/Oncology, Neurology, and Gastroenterology. Critical Care/Surgery included Emergency Medicine, Anesthesia, Pulmonary Medicine, Surgery (General Surgery, Vascular Surgery, Urology, Orthopedics), and Obstetrics/Gynecology. Within the 38 papers, 5 papers were descriptive, summarizing the general perspectives of program directors, faculty and/or trainees related to AHD. Thirty-three papers represented a specific AHD educational experience and were evaluated further. Table 1 summarizes these background characteristics.

**Table 1:** Summary of 38 papers included in academic half-day scoping review: background characteristics.

**Characteristic**	**Range (median) or Number (%)**
**Paper publication year**	1977–2019 (2014)
**Paper country**	
United States	19 (50)
Canada	19 (50)
**Paper discipline**	
Medicine^*^	17 (45)
Pediatrics	10 (26)
Critical Care/Surgery^**^	9 (24)
Radiology	2 (5)
**Paper focus**	
Specific educational experience	33 (87)
General perspectives (descriptive)	5 (13)

* *Medicine includes Geriatrics, Family Medicine, Hematology/Oncology, Neurology, Gastroenterology*.

** *Critical Care/Surgery includes Emergency Medicine, Anesthesia, Pulmonary Medicine, Surgery (General Surgery, Vascular Surgery, Urology, Orthopedics), and Obstetrics/Gynecology*.

For the 33 specific educational experience papers, one described 3 separate AHD educational experiences, totaling 35 AHD studies. Table 2 summarizes learner and teaching characteristics of these 35 AHD studies. Most papers focused only on resident learners (*n* = 27, 77%). We found descriptions of various teaching strategies. The large majority of studies (*n* = 31, 89%) described educational experiences that employed interactive approaches to teaching, like simulation, case-based learning, problem-based learning, or role play, often combined with didactics. Four studies (11%) described AHD educational experiences that employed didactics exclusively. The numbers of learners participating in the AHD experience ranged from 5 to 364 (median 39). AHD length was 1.5–6 h (median 3).

**Table 2:** Summary of 33 papers (35 studies) addressing specific academic half-day experiences: learner and teaching characteristics.

**Characteristic**	**Range (median) or Number (% of 35 studies)**
**Learner population**	
Residents only	27 (77)
Fellows only	1 (3)
Faculty only	1 (3)
Combined groups (residents, fellows, faculty)	6 (17)
**Learner numbers**	5–364 (39)
**Teaching strategies**	
Multi-modal, including didactics	16 (46)
Case-based learning + other	6 (17)
Simulation + other	5 (14)
Didactics only	4 (11)
Simulation only	2 (6)
Problem-based learning only	1 (3)
Role play only	1 (3)
**Teaching time (hours)**	1.5–6.0 (3)

All 33 papers described a rationale for pursuit of the medical education research study. In three papers, the authors wanted to directly assess the AHD educational format by collecting data that represented AHD effectiveness (10, 19, 20). In six papers, authors pursued an interest in how AHD compared to a traditional curriculum of several hourly educational sessions per week (3, 11, 12, 21–23). In the remaining 24 papers, authors were prompted by concerns about inadequate formal curricula on certain topics or skills (e.g., communication skills, interviewing skills, specialty-specific clinical management, anatomic understanding to improve performance of clinical procedures) (24–47).

Thirty-two of 35 AHD studies (91%) described AHD teachers; many AHD experiences included trainees as teachers. Of the other resources that are required for delivery of AHD–space, funding, technology, equipment, standardized patients–some were noted or described in 25 studies (71%), but not with consistency. No papers compared resources needed for AHD-related teaching to resources needed for a comparable duration of traditional 1-h teaching (e.g., daily noon conferences).

All of the 35 AHD studies represented a medical education research project that, in some fashion, evaluated the effect of the AHD experience on learners. A minority of studies (*n* = 4) used rigorous research designs [randomized controlled trial (RCT) (*n* = 2); 2 groups pre/post (*n* = 2)]. In contrast, the majority of studies (*n* = 31) used weaker research designs [1 group, pre/post (*n* = 19); 1 group, post only (*n* = 8); 2 groups, post only (*n* = 4)]. Potential effects on learner outcomes were measured across three of the four levels described by the Kirkpatrick Model of Training Evaluation, including Level 1 “reaction” (27 studies), Level 2 “learning” (30 studies), and Level 3 “behavior” (12 studies) (16). There were no studies addressing the effect of the AHD experience on the highest level of the Kirkpatrick model: Level 4 “results”, such as patient-level outcomes.

Of the 31 studies using weaker research designs, almost all concluded that the AHD experience had positive effects (see Table 3). Of the four studies using stronger research designs, results were mixed. One study showed more positive effects of AHD experience compared to control experience (i.e., on self-reported attitudes and observed clinical skills) (25). One study showed more positive effects of AHD experience using high-fidelity simulation mannequin compared to AHD experience using low-technology mannequin (i.e., on observed clinical skills) (31). One study reported similar effects of AHD experience and control experience (i.e., on observed clinical skills) (36). Finally, one study showed fewer positive effects of AHD experience compared to control experience (i.e., on long-term knowledge retention) (21).

**Table 3:** Summary of 35 studies using weaker and stronger research designs, with description of academic half-day outcomes, by Kirkpatrick level.

	**Weaker research design (*****n*** **=** **31)**		**Stronger research design (*****n*** **=** **4)**	
**Outcomes**	**Outcome measured Number of papers**	**If outcome measured, positive effect reported *N* (%)**	**Outcome measured Number of papers**	**If outcome measured, differential positive effect peported *N* (%)**
**Kirkpatrick level 1–Learner reaction**				
Attendance	6	6 (100)	0	0 (0)
Interruption	4	4 (100)	0	0 (0)
Satisfaction	27	26 (96)	0	0 (0)
**Kirkpatrick level 2–Learner learning**				
Immediate knowledge	20	19 (95)	2	1 (50)
Sustained knowledge	7	6 (86)	1	1 (100)
Skills	8	6 (75)	1	1 (100)
Attitudes	22	22 (100)	1	1 (100)
**Kirkpatrick level 3–Learner behavior**				
Application to clinical practice	12	12 (100)	2	1 (50)
**Kirkpatrick level 4–Results**				
Patient improvement	0	0 (0)	0	0 (0)

## Discussion

We conducted this scoping review to describe key characteristics of the AHD teaching method as used in Post-graduate medical education curricula and to understand its potential effect on Post-graduate medical trainees. In summary, we found that the majority of papers on AHD were published in or after 2014, originating from either Canada or the United States, delivered primarily to resident learners, and most employed interactive and multi-modal teaching methods. Other reported AHD characteristics (e.g., length, number of learners) varied widely. Few studies used rigorous medical education research designs. Compared to lower level Kirkpatrick outcomes, like learner satisfaction and learner knowledge/attitudes, there has been less frequent assessment of higher level Kirkpatrick outcomes such as learner clinical skills or patient improvement/clinical outcomes.

### Results in Context

Post-graduate training programs increasingly have turned to the use of AHD as an alternate educational model. Historically, the AHD teaching model appears to have first been utilized in Canada (13) and has gradually gained implementation in the US (10, 11). As the review highlighted, in the pre-2014 period, there were 7 papers self-described as AHD-focused (Canada = 6; US = 1) and in the after-2014 period, there were 16 papers self-described as AHD-focused (Canada = 9; US = 7). The Canada and US traditionally have similar structures for resident and fellowship training. The AHD may be more feasible in this type of training model, different from other English-speaking countries, which, in addition to our exclusion of papers not written in English, may explain this apparent regional preference, and why our scoping review did not identify papers on this topic from other countries. We assume that the number of studies of AHD is roughly reflective of the use of AHD in Post-graduate medical education, and while this review spanned ~42 years, our finding that most studies were published during the last 6 years, is an indicator of the increasing popularity of the AHD teaching model.

Our scoping review results revealed that descriptions of individual AHDs in Post-graduate medical education curricula varied widely with regard to duration and number of learners. We acknowledge that the range of duration of AHD sessions (1.5–6 h) that we found may have been influenced by our inclusion/exclusion criteria, namely that the teaching had to be described as a single intensive learning event (i.e., more than 1 h), but could not be a full or multi-day lecture/training. We chose to include educational experiences lasting more than 1 h, to distinguish them from the traditional 1-h noon conference, but excluded those that were full-day or more, as this type of educational experience has quite different implications for administration (particularly in regard to scheduling and coverage of patient care). While the AHD affords an extended protected opportunity for educational time, trainees can still attend to necessary clinical responsibilities during other parts of the day, which would not necessarily be possible with a full-day educational experience. It was interesting to note that the number of learners varied so widely, from intimate groups to large forums, since this may have also influenced the types of teaching modalities that were feasible.

In this review, the AHD target audience/learner group was predominantly residents, as opposed to other Post-graduate medical trainees, like fellows. Classes of residents are typically larger than classes of fellows. Residents are less likely than fellows to have frequent and in-depth experiences with faculty. Residents are also frequently widely dispersed with regards to their clinical duties and physical locations. For these reasons, residents may represent a trainee learner group for which a block of educational time is more attractive than a dispersed teaching model. This hypothesis is supported by many of the stated rationales for trying out the AHD model. In 24 papers, the rationale for a change in learner experience was a need to address concerns about inadequate formal curricula on specific topics or skills. In six other papers, the stated rationale was an interest in how AHD compared to a traditional curriculum of several hourly educational sessions per week (such as resident noon conferences).

Our review results showed that individual AHDs varied with regards to the use of different educational modalities. Evaluation of extended interactive case-based learning shows that, when compared to traditional didactic experiences, it results in significantly improved resident attendance, lowered distractibility, and improved resident satisfaction (12). It would be interesting to compare levels of effectiveness of AHD in studies that use mixed interactive methods, including role plays, simulations, and/or case-based learning, versus studies that depend almost exclusively on didactic presentations.

We noted that papers primarily described who delivered the curriculum but did not consistently or fully describe the other resources needed to implement the AHD. For example, almost all studies described AHD-dedicated personnel resources, while other required resources (e.g., space, technology, standardized patient, funding) were less consistently described. This information is important for decision-making by Program Directors or others tasked with developing curriculum for Post-graduate medical trainees.

All 35 studies, in the 33 full-text papers, described a medical education research project that evaluated the effect of the AHD experience on learners. While this finding is very encouraging, the majority were limited by weak research designs, and outcomes solely measured by learner self-report. There were four studies using stronger designs (RCT or Non-randomized 2-group pre/post) (21, 25, 31, 36). The Abraham et al., RCT study showed more positive learner outcomes for AHD compared to a reading assignment (25). The Donoghue et al., RCT study highlighted the importance of AHD teaching components, showing that there were more positive learner outcomes for AHD with high-fidelity simulation mannequin compared to AHD with low-tech mannequin (31). The Farsad et al., 2-group study indicated no difference in observed interviewing skills for learners in “AHD” study group vs. control group (36). The Raman et al., 2-group study showed that long-term knowledge retention was less for AHD massed learning than for dispersed learning (21). Thus, though the number of higher-quality studies was small, the results were mixed.

Variation in AHD design and individual study choice and presentation of outcome measures precluded a systematic review of this literature. Instead of presenting each specific learner outcome, in keeping with the role of the scoping review as a way to identify potential gaps in existing literature and research, we explicitly chose to present an overview of learner outcomes using the Kirkpatrick Model of Training Evaluation framework. Most studies evaluated the AHD at the Kirkpatrick Level 1 “reactions” and Level 2 “learning”; fewer studies assessed Level 3 “behavior” and no studies assessed Level 4 “results”. Ultimately, when designing educational curricula, we strive to implement educational approaches that positively affect learner-level clinical skills and patient-level clinical outcomes (Level 3 and 4). Our review results identify a gap in the literature regarding the evaluation and effect of AHD on these higher-level learner outcomes.

### Recommendations

AHD is becoming increasingly popular in Post-graduate medical education curricula. However, the findings of our scoping review demonstrate less variety in published studies on this topic with regards to target learner population or teaching strategies. In contrast, considerable variability was found with regards to time allotted for the delivery of the AHD educational experience and number of participants.

We suggest that medical educators should learn a lot more about the AHD before making a commitment to transition to this teaching method on a large scale, especially since the level of resources required to develop, organize, and deliver an AHD curriculum has been so sparsely described. Given the widely varied descriptions of the individual AHDs, we would encourage the conceptualization and creation of a standardized definition of an AHD (i.e., length, frequency, size, multiple topics covered vs. immersed learning in one topic, resources required etc.). Publication of studies on AHD should require systematic description about these features of the AHD, in order to guide medical educators in the development of new AHD curriculum, and to promote a shared understanding of the AHD as a unique and specific type of educational experience. This standardization will also ensure more meaningful conclusions of future AHD reviews and research than is currently possible.

In order to comprehend the breadth of the AHD model, it is necessary to conduct high-quality research that (1) assesses which AHD teaching strategies have larger effects, relative to learning purpose, contents/topics, and learner training level [see Donoghue et al. (18) as an example]; and (2) compares the AHD experience to other frequently used teaching models. While measured outcomes of AHD educational experiences showed positive effects on lower Kirkpatrick level learner outcomes like satisfaction and knowledge, there is a paucity of research describing the higher Kirkpatrick level effects of AHD on outcomes related to learner application of the educational experience to clinical practice and/or patient outcomes. We would encourage medical education researchers interested in AHD development to consider including Level 4 “results” or patient-level outcome measures in their study design. Depending on the content of the AHD, this might be achievable by collecting relevant quantitative (e.g., change in prevalence or severity of condition, change in markers of patient control of condition) and/or qualitative data (e.g., patient perceived experience, patient sense of control of condition). The outcome should be time limited, so that it is measured in close enough proximity to the educational experience to consider possible impact. Additionally, the majority of studies utilized weaker research designs, and the effects of AHD were found to be mixed in those few studies that used stronger research designs. Further investigation of AHD that employs rigorous research designs and includes measured clinical application and patient outcomes is necessary to improve the quality of the science and increase our understanding.

### Strengths and Limitations

The main strength of the present study is the use of scoping review methodology with a five-stage framework applied from Arksey and O'Malley, maximizing transparency and reproducibility. A limitation of this study is the exclusion of studies written in Non-English languages, and as such, some educational experiences may not be included. We could have limited our study to only papers that “self-describe as an AHD” educational experience which might have provided us a better depiction of characteristics of the AHD teaching strategy.

## Conclusions

In summary, we conducted a scoping review on AHD teaching model in Post-graduate medical training. We were able to examine descriptions of individual AHD educational experiences, as well as explore measured educational outcomes of the curricula. The data collected and presented in this study represent the beginning of a deep understanding of the characteristics and potential importance of the AHD teaching model in Post-graduate medical education. The results highlight specific knowledge gaps, suggesting that further primary research is needed. Additional studies with rigorous study designs, carefully defined and described educational programs, and measurement of clinical patient outcomes, could assist with a more reliable understanding of the AHD and inform the planning, development, and implementation of future AHD curricula. After the conduct of additional and rigorous medical education research in this area, a logical next step will be a systematic review with meta-analysis used to aggregate results.

## Data Availability Statement

The original contributions presented in the study are included in the article/Supplementary Material, further inquiries can be directed to the corresponding author/s.

## Author Contributions

MC, LCH, HF, and LMH contributed to the conception of manuscript aims/research questions, identifying relevant databases and key terms, and establishing inclusion and exclusion criteria. MC and LMH completed title/abstract screening, full text eligibility review of all papers, developed a data-charting form, and performed data abstraction of eligible full text papers. LCH resolved disagreements about study inclusion or exclusion at each step of eligibility assessment. MC, LCH, and LMH interpreted and analyzed data-charting form. All authors contributed to the development of discussion and conclusions of manuscript. All authors were major contributors in the writing and editing of the manuscript. All authors read and approved the final manuscript.

## Funding

MC was supported by the HRSA MCHB DBP Training Program (T77MC09796) awarded to the Stanford University Developmental-Behavioral Pediatrics Fellowship Program.

## Conflict of Interest

The authors declare that the research was conducted in the absence of any commercial or financial relationships that could be construed as a potential conflict of interest.

## Publisher's Note

All claims expressed in this article are solely those of the authors and do not necessarily represent those of their affiliated organizations, or those of the publisher, the editors and the reviewers. Any product that may be evaluated in this article, or claim that may be made by its manufacturer, is not guaranteed or endorsed by the publisher.

## Abbreviations

AHD: academic half-day
ACGME: Accreditation Council for Graduate Medical Education
NC: noon conference
RCT: randomized controlled trial.
