# Supplementary material for: Academic Half-Day Education Experience in Post-graduate Medical Training: A Scoping Review of Characteristics and Learner Outcomes
Source: Front Med (Lausanne). 2022 Mar 2;9:835045. doi: 10.3389/fmed.2022.835045 (PMC8926071; doi:10.3389/fmed.2022.835045)
Supplement: Supplementary file 1 [file Data_Sheet_1.docx]

**Supplementary Material 1**

**Main Concepts and Database Search Strategies**

Search strategies created and executed by Myong Sun Choe, MD, September 13, 2019

Total results = 735

**PubMed.** (“academic half day” [tw] OR “academic half days” [tw] OR “noon conference” [tw] OR “noon conferences” [tw] OR (“block” [ti] AND conference” [ti]) OR (“noontime [ti] AND conference* [ti]) OR “didactic sessions” [tw] OR “didactic session” [tw] OR “immersion week” [tw] OR “immersion weeks” [tw] OR “intensive blocks” [tw] OR “intensive block” [tw]) AND (“internship and residency” [mesh] OR “clinical fellows” [tw] OR “clinical fellow” [tw] OR fellow* [tw] OR interns [tw] OR residents [tw] OR postgraduate* [tw] OR residency [tw]) AND english [lang]

= 302 results.

**Web of Science.** (“academic half day” OR “academic half days” OR “noon conference” OR “noon conferences” OR (“block” AND conference” ) OR (“noontime AND conference* ) OR “didactic sessions” OR “didactic session”  OR “immersion week”  OR “immersion weeks” OR “intensive blocks” OR “intensive block”) AND (“clinical fellows” OR “clinical fellow” OR fellow* OR interns OR residents OR postgraduate* OR residency)

= total 300 results.

**Education Resources Information Center (ERIC).** "academic half day" OR "academic half days" OR "noon conference" OR "noon conferences" OR (block AND conference) OR (noontime AND conference*) OR "didactic sessions" OR "didactic session" OR "immersion week" OR "immersion weeks" OR "intensive blocks" OR "intensive block"

= total 26 results.

**MedEdPORTAL.**

“noon conference” = 11 results.

“academic half days” = 1 result.

“academic half day” = 13 results.

= total 25 results.

**Google Scholar (from first 2 pages of search).**

“noon conference” site:.org = 16 results.

“noon conference” site:.edu = 9 results.

“noon conference” filetype:pdf = 8 results.

“academic half days” site:.org = 14 results.

“academic half days” site:.edu = 0 result.

“academic half days” filetype:pdf = 0 result.

“academic half day” site:.org = 15 results.

“academic half day” site:.edu = 3 results.

“academic half day” filetype:pdf = 2 results.

= total 67 results.

**Google (from first 2 pages of search).**

“noon conference” OR “academic half days” OR “academic half day” = total 15 results.
