# Supplementary material for: Academic Half-Day Education Experience in Post-graduate Medical Training: A Scoping Review of Characteristics and Learner Outcomes
Source: Front Med (Lausanne). 2022 Mar 2;9:835045. doi: 10.3389/fmed.2022.835045 (PMC8926071; doi:10.3389/fmed.2022.835045)
Supplement: Supplementary file 2 [file Data_Sheet_2.docx]

**Supplementary Material 2**

**Study Characteristics of AHD Teaching Strategies**

| **Authors, year** | **Country** | **Paper Type**  **Self-described AHD (Y/N)** | **Discipline** | **Target Audience** | **# Participants** | **Rationale** | **Delivery Time (hr)** | **Teaching Strategies** | **Resources (personnel)** | **Resources (other: space, money, SP, etc.)** | **Evaluation Design** | **Learner Outcome / Kirkpatrick Level** |
| --- | --- | --- | --- | --- | --- | --- | --- | --- | --- | --- | --- | --- |
| Abbott et al., 2014^1^ | Canada | Specific Educational experience + Evaluation  N | Family medicine | Resident | 69 | Residents should use lab tests more efficiently and appropriately. | 4 | Didactics | General pathologist and 2 senior general pathology residents | Classroom, facility tours, pre-session slide presentation | One group, pre/post | 1, 2 |
| Abraham et al., 2001^2^ | US | Specific Educational experience + Evaluation  N | Adolescent medicine | Resident | 56 | Few evaluations of violence prevention education programs exist. | 3 | Multi-modal, incl didactics (Didactics + SP + Role play) | Teen peer educators | SP | RCT  Two groups, pre/post | 2, 3 |
| Aggarwal et al., 2012^3^ | US | Specific Educational experience + Evaluation  N | Anesthesia | Resident | 24 | More anesthesia providers need training in speciﬁc skills required for perioperative care of liver transplant patients. | 4 | Multi-modal, incl didactics (Didactics + SP + Simulation) | Central Line/Torso task trainer | SP  Online materials, simulation mannequin, anesthesia machine, standard operating room monitors | One group, pre/post | 1, 2 |
| Audcent et al., 2013^4^ | Canada | Specific Educational experience + Evaluation  Y | Pediatrics | Resident | 125 | Opportunities for resident global health training and exposure are most often limited to electives or trainees in dedicated global health tracks. | Two 2-hour sessions | Multi-modal, incl didactics (Didactics + Role play + Video) | 2 “site champion” global health enthusiasts | Comprehensive trainer manual, slides including video clips | One group, pre/post | 1, 2, 3 |
| Batalden et al., 2013^5^ | US | Specific Educational experience +Evaluation  3 educational experiences (different sites)  Y | Internal medicine | 1) Resident/Faculty  2) Resident/Faculty  3) Faculty | 1) 39  2) 122  3) 135 | There is limited data on AHD. | 1) 4  2) 2.5  3) 1.5 | 1) CBL + Other (Role play/simulation)  2) CBL + Other (Didactic + Skills-based exercises)  3) CBL + Other (Team-based learning) | 1) Associate program director; subspecialty faculty members; clinical coverage  2) Program director; 5 chief residents; clinical coverage  3) 5 associate program directors; 1 chief resident; clinical coverage | 1) study materials; computer work stations  2) textbooks; teaching materials  3) teaching materials; Clicker audience response system, 6 touchscreen tablets, wireless booster antenna; | 1) Residents - One-group, pre/post (perception) and post (ITE)  Faculty – one-group, post  2) Residents - One-group, pre/post (self-report) and post (ITE)  Faculty – one group, post (anecdotal)  3) Faculty – one group, post (anecdotal) | 1) 1, 2  2) 1, 2  3) 1 |
| Benson et al., 2018^6^ | US | Specific Educational experience + Evaluation  N | Family medicine | Resident | 71 | Resident education about awareness of healthcare disparities in the learning environment should be strengthened. | 1.2 | PBL | Residents | Not reported | One group, pre/post | 1, 2, 3 |
| Chesney et al., 2018^9^ | Canada | Specific Educational experience + Evaluation  N | Surgery | Resident | 18 | Surgeons lack training in preference-sensitive communication skills to provide decisional support in situations that require complex weighing of desirable and undesirable outcomes. | 2 | Multi-modal, incl didactics (Didactics + Role play) | 2 faculty | Not reported | One group, pre/post and 6 months post | 1, 2, 3 |
| Denizard-Thompson et al., 2018^10^ | US | Specific Educational experience + Evaluation  Y | Internal medicine | Resident | 93 | Internal medicine residents report low confidence in performing musculoskeletal examinations and intra-articular steroid injections. | 2.8 | Simulation + other (Simulation + Didactics + Game + Online resources) | 1 or 2 clinicians (chief resident, educational liaison, program director) | Not reported | One group, pre/post | 1, 2 |
| Di Genova et al., 2015^11^ | Canada | Specific Educational experience + Evaluation  Y | Pediatrics | Resident | 18 | Redesigned its AHD based on program concerns including inadequate preparation for general pediatric practice, gaps in CanMEDS education and exclusive use of didactic lectures. | 4 | Simulation + other (Simulation + Didactics) | Division directors, general pediatricians | Financial remuneration | One group, post | 1, 2, 3 |
| Donoghue et al., 2009^12^ | Canada | Specific Educational experience + Evaluation  N | Pediatrics | Resident | 51 | Residents have scant experiences in leading resuscitations for pediatric patients. | 1.5 | Simulation + other (Simulation + Didactics + CBL) | Investigator | Not reported | RCT  Two groups, pre/post | 2 |
| Downar et al., 2012^13^ | Canada | Specific Educational experience + Evaluation  N | Critical care medicine | Resident, Fellow | 51 | Few training programs teach communication skills in a formal way to deliver complicated medical information or bad news to distressed patients and family members, and participate in making difficult decisions in an ethically sound manner. | 4 | Multi-modal, incl didactics (Didactics + SP) | Faculty member or trained mediator | SP | One group, pre/post and 1-4 years post | 1, 2 |
| Dumont et al., 2014^14^ | Canada | Specific Educational experience + Evaluation  Y | OB/GYN | Resident | 24 | Residents receive limited exposure to the concepts and techniques associated with PAG. | 3 | Simulation | Specialist in Pediatric and Adolescent Gynecology | Not reported | One group, post | 1, 2 |
| Dumont et al., 2016^15^ | Canada | Specific Educational experience + Evaluation  Y | OB/GYN | Resident | 35 | Residents do not always graduate feeling comfortable with the PAG population. | 3 | Simulation | Specialist in PAG | Simulation center, advanced pelvic models for procedure-specific stations | One group, pre/post | 2 |
| Eid et al., 2015^16^ | US | Specific Educational experience + Evaluation  Y | Hematology/Oncology | Fellow | Not reported | Few published studies have evaluated the effectiveness of changing the traditional curriculum of several hourly educational sessions per week to an AHD educational format. | 3-4 | Didactics | Not reported | Not reported | One group, post | 1, 2 |
| Eisenberg et al., 2019^17^ | US | Specific Educational experience + Evaluation  Y | Internal medicine | Resident | 19 | Little is known about effective strategies to teach medical trainees to respond to discrimination by patients. | 3 | Multi-modal, incl didactics (Didactics + SP) | Faculty member, 4 senior and chief resident facilitators | SP  Patient safety training center | One group, pre/post | 1, 2 |
| Farsad et al., 1978^19^ | US | Specific Educational experience + Evaluation  N | Pediatrics | Resident | 39 | No information is available on the interviewing skills of different levels of pediatric trainees and practicing pediatricians. | 1.5 | Didactics | Not reported | SP | Two groups, pre/post | 3 |
| Ha et al., 2014^20^ | US | Specific Educational experience + Evaluation  Y | Internal medicine | Resident | 364 | Little is known about AHD curriculum regarding its effect on knowledge acquisition and resident satisfaction. | 3 | Multi-modal, incl didactics (Didactics + CBL) | Not reported | Lunch | Two groups, post | 1, 2 |
| Harrison et al., 1999^21^ | US | Specific Educational experience + Evaluation  N | Emergency medicine | Resident | 20 | Emergency physicians should be prepared to provide impartial, objective testimony in legal proceedings. | 2 | Multi-modal, incl didactics (Didactics + CBL) | 1 attorney-at-law per 10 residents | Large conference room | One group, pre/post | 2 |
| Miller et al., 2016^22^ | US | Specific Educational experience + Evaluation  N | Radiology | Resident | 61 | Teaching principles of patient-centered healthcare delivery and patient interaction in a radiology residency setting is often difficult and ineffective in a traditional lecture format. | 1.5 | CBL | Program directors | Conference room, tables, chairs, anonymized actual patient letters, Survey Monkey, audio recording application on a standard tablet computer | One group (at 2 different institutions), post | 1, 2, 3 |
| Moreno et al., 2013^23^ | US | Specific Educational experience + Evaluation  N | Pediatrics | Resident | 70 | Resident attendance and participation at didactic conferences is often limited owing to time demands. | 3.5 | Multi-modal, incl didactics (Didactics + CBL) | 2 faculty and 1 resident per session | $5 gift card | Two groups, post | 1, 2 |
| Pembroke et al., 2017^24^ | Canada | Specific Educational experience + Evaluation  Y | Radiation oncology | Resident | 10 | QI skills must be taught and formally established within Radiation Oncology residents' curriculum. | 2 X 6 (sessions)  3 (online) | Multi-modal, incl didactics (Didactics + Workshops + Self-directed online modules) | Clinical fellow, 3 staff physicians, expert in QI methods | Online training using the Institute of Healthcare Improvement and Healthcare QI Partnership teaching programs | One group, pre/post | 1, 2 |
| Raman et al., 2010^25^ | Canada | Specific Educational experience + Evaluation  Y | Gastroenterology | Resident | 20 | There is limited data whether didactic or interactive educational delivery methods among physicians can achieve similar results on long-term retention of knowledge. | 4 | Didactics | 1 GI physician nutrition expert | Not reported | Two groups, pre/post and delayed post | 2 |
| Ramar et at., 2015^26^ | US | Specific Educational experience + Evaluation  N | Pulmonary, Critical Care | Resident, Fellow | 7 | QI tools are essential to be taught in medical school, residency, and fellowship training programs to improve quality and safety in patient care. | 2-hour of video and a 4-hour of AHD session | Multi-modal, incl didactics (Flipped classroom with didactics (home) + PBL (class)) | 2 QI instructors, 5 physicians, faculty moderator | REDCap | One group, pre/post | 1, 2 |
| Reznek et al., 2003^27^ | US | Specific Educational experience + Evaluation  N | Emergency medicine | Resident | 13 | There is no established curriculum that teaches the necessary crisis management skills in emergency care. | 5-6 | Simulation + other incl didactics (Simulation + Didactics) | Residency director | SP  Simulated ED, simulation mannequin | One group, post | 1, 2 |
| Robbins et al., 2018^28^ | US | Specific Educational experience + Evaluation  Y | Vascular surgery | Resident, Fellow | 6 | Work-hour restrictions, patient care duties, and operative schedules create barriers for surgical trainees to attend didactics. | 3 | Multi-modal, incl didactics (Didactics + Conference discussion) | 7 vascular surgery faculty | Not reported | One group, pre/post | 1, 2 |
| Schinasi et al., 2018^29^ | US | Specific Educational experience + Evaluation  N | Pediatrics | Resident | 27 | Immersive simulation provides physicians with the opportunity to practice complex skill to recognize when a patient safety events has occurred and effectively carry out disclosure, all while caring for a patient who is actively experiencing the consequences of an unintended outcome. | 3 | Simulation + other (Simulation + Didactics + Interactive Small Group Discussion) | 6 core faculty members (both physicians and nurses), lead facilitator, 2 confederates (nurse, parent), 1 behind-the-scenes simulation technician | Simulated pediatric patient + confederate parent/nurse.  Simulation center. Estimated cost for 27 residents was $6,993, not accounting for 39 per clinician facilitator. | One group, post and delayed post | 1, 2, 3 |
| Stokes et al., 2017^30^ | Canada | Specific Educational experience + Evaluation  Y | Internal medicine | Resident | 207 | Lack of formal teaching sessions are barriers to the success in Guyana-based, internal medicine post-graduate medical education program. | 3 | CBL | Medical residents (weekly teaching). Administrative staff. Information technologist support. | Videoconferencing equipment | Two groups, post | 1, 2, 3 |
| Tam et al., 2017^32^ | Canada | Specific Educational experience + Evaluation  Y | Pediatrics | Resident | 28 | Teaching resources for CBL in pediatric infectious diseases are limited. | 1-1.5 | CBL | Attending physician, fellow, senior resident | Module composed of two resource files, case file, suggested reading list | Two groups, post | 1, 3 |
| Wong et al., 2007^34^ | Canada | Specific Educational experience + Evaluation  Y | Internal medicine | Resident | 72 – 81 | Operational delivery of formal curriculum for residency training in internal medicine has been challenging for many postgraduate programs. | 4 | Multi-modal, incl didactics (Didactics + Physical Exam + Workshops + CBL) | Chief medical residents, AHD organizing committee (e.g., chief residents, class representatives, APD) | Not reported | One group, post | 1 |
| Wong et al., 2009^35^ | Canada | Specific Educational experience + Evaluation  Y | Internal medicine | Resident | 115 | Challenges remain in addressing effective patient-physician communication in medical education. | 4 | Multi-modal, incl didactics (Didactics + Interactive session + Video excerpts + Interactive reflection) | AHD director, faculty members, senior residents, chief medical residents | Personal response system | One group, pre/post | 1, 2 |
| Yuasa et al., 2013^36^ | US | Specific Educational experience + Evaluation  N | Geriatric, Family medicine | Resident, Fellow, Faculty | 62 | Little formal training is provided for healthcare professionals how to effectively handle telephone calls about nursing home residents. | 2-3 | Role play | GM faculty, FM faculty, NP faculty | Not reported | One group, pre/post | 2, 3 |
| Zastoupil et al., 2017^37^ | US | Specific Educational experience + Evaluation  Y | Pediatrics | Resident | 32 | No studies have utilized rigorous qualitative methods to better understand its impact and resident experiences with AHD. | 3.5 | Multi-modal, incl didactics (Didactics + CBL or Questions or Games) | 1 resident APD | Software | One group, pre/post (survey) and post (focus groups) | 1, 3 |
| Zeller et al., 2015^38^ | Canada | Specific Educational experience + Evaluation  Y | Internal medicine | Resident | 31 | Internal medicine programs rarely incorporate anatomy review into clinical procedural teaching. | 3 | Multi-modal, incl didactics (Didactics + Interactive Lecture +  Video and visualization exercise + SP) | Hematology staff consultants | SP | One group, pre/post | 2 |
| Chalk, 2004^7^ | Canada | Descriptive Survey  Y | Neurology | Program director | 21 | There are few hard data about the prevalence, content, or structure of the AHD in Canadian residency programs, including those in child and adult neurology. | N/A | N/A | N/A | N/A | N/A | N/A |
| Chen et al., 2015^8^ | Canada | Descriptive Survey  Y | Internal medicine, orthopedic surgery, hematology | Resident | 27 | There is remaining question about what role classroom-based education in the form of AHD has in the resident’s learning experiences. | N/A | N/A | N/A | N/A | N/A | N/A |
| Evidence- Based Medicine Working Group, 1992^18^ | Canada | Descriptive Survey  Y | Internal medicine | Resident | N/A | Evidence-based medicine requires new skills of the physician, including efficient literature searching and the application of formal rules of evidence evaluating the clinical literature. | N/A | N/A | N/A | N/A | N/A | N/A |
| Tagger et al., 2018^31^ | Canada | Descriptive Survey  Y | Urology | Resident | 190 | Residency experiences and teaching in oncology among urology residents are variable across Canada. | N/A | N/A | N/A | N/A | N/A | N/A |
| Wagoner et al., 2019^33^ | US | Descriptive Survey  Y | Pediatrics | Attending | 15 | An understanding of the impact of AHD on attending physicians and patient care has not been well described. | N/A | N/A | N/A | N/A | N/A | N/A |

Abbreviations: AHD, Academic Half-day; APD, associate program director; CBL, case-based learning; ED, emergency department; GI, gastroenterology; GM, general medicine; FM, family medicine; N, no - not self-identified as AHD; N/A, not applicable; NP, nurse practitioner; PAG, pediatric and adolescent gynecology; PBL, problem-based learning; QI, quality improvement; RCT, randomized controlled trial; SP, standardized patient; US, United States; Y, yes - self-identified as AHD;

1. Abbott M, Paulin H, Sidhu D, Naugler C. Laboratory tests, interpretation, and use of resources: a program to introduce the basics. *Can Fam Physician.* 2014;60(3):e167-172.

2. Abraham A, Cheng TL, Wright JL, Addlestone I, Huang Z, Greenberg L. Assessing an educational intervention to improve physician violence screening skills. *Pediatrics.* 2001;107(5):E68.

3. Aggarwal S, Bane BC, Boucek CD, Planinsic RM, Lutz JW, Metro DG. Simulation: a teaching tool for liver transplantation anesthesiology. *Clin Transplant.* 2012;26(4):564-570.

4. Audcent TA, Macdonnell HM, Moreau KA, et al. Development and evaluation of global child health educational modules. *Pediatrics.* 2013;132(6):e1570-1576.

5. Batalden MK, Warm EJ, Logio LS. Beyond a curricular design of convenience: replacing the noon conference with an academic half day in three internal medicine residency programs. *Acad Med.* 2013;88(5):644-651.

6. Benson BL, Ha M, Stansfield RB, Markova T. Health Disparities Educational Initiative for Residents. *Ochsner J.* 2018;18(2):151-158.

7. Chalk C. The academic half-day in Canadian neurology residency programs. *Can J Neurol Sci.* 2004;31(4):511-513.

8. Chen LY, McDonald JA, Pratt DD, Wisener KM, Jarvis-Selinger S. Residents' views of the role of classroom-based learning in graduate medical education through the lens of academic half days. *Acad Med.* 2015;90(4):532-538.

9. Chesney T, Devon K. Training surgical residents to use a framework to promote shared decision-making for patients with poor prognosis experiencing surgical emergencies. *Can J Surg.* 2018;61(2):114-120.

10. Denizard-Thompson N, Feiereisel KB, Pedley CF, Burns C, Campos C. Musculoskeletal Basics: The Shoulder and the Knee Workshop for Primary Care Residents. *MedEdPORTAL.* 2018;14:10749.

11. Di Genova T, Valentino PL, Gosselin R, Bhanji F. The Academic Half-Day redesigned: Improving generalism, promoting CanMEDS and developing self-directed learners. *Paediatr Child Health.* 2015;20(1):30-34.

12. Donoghue AJ, Durbin DR, Nadel FM, Stryjewski GR, Kost SI, Nadkarni VM. Effect of high-fidelity simulation on Pediatric Advanced Life Support training in pediatric house staff: a randomized trial. *Pediatr Emerg Care.* 2009;25(3):139-144.

13. Downar J, Knickle K, Granton JT, Hawryluck L. Using standardized family members to teach communication skills and ethical principles to critical care trainees. *Crit Care Med.* 2012;40(6):1814-1819.

14. Dumont T, Hakim J, Black A, Fleming N. Enhancing postgraduate training in pediatric and adolescent gynecology: evaluation of an advanced pelvic simulation session. *J Pediatr Adolesc Gynecol.* 2014;27(6):360-370.

15. Dumont T, Hakim J, Black A, Fleming N. Does an Advanced Pelvic Simulation Curriculum Improve Resident Performance on a Pediatric and Adolescent Gynecology Focused Objective Structured Clinical Examination? A Cohort Study. *J Pediatr Adolesc Gynecol.* 2016;29(3):276-279.

16. Eid A, Hsieh P, Shah P, Wolff R. Cross-sectional longitudinal study of the academic half-day format in a hematology-oncology fellowship training program. *BMC Med Educ.* 2015;15:139.

17. Eisenberg EH, Kieffer KA. Use of Simulated Patient Encounters to Teach Residents to Respond to Patients Who Discriminate Against Health Care Workers. *J Gen Intern Med.* 2019;34(5):764-768.

18. Evidence-Based Medicine Working G. Evidence-based medicine. A new approach to teaching the practice of medicine. *JAMA.* 1992;268(17):2420-2425.

19. Farsad P, Galliguez P, Chamberlin R, Roghmann KJ. Teaching interviewing skills to pediatric house officers. *Pediatrics.* 1978;61(3):384-388.

20. Ha D, Faulx M, Isada C, et al. Transitioning from a noon conference to an academic half-day curriculum model: effect on medical knowledge acquisition and learning satisfaction. *J Grad Med Educ.* 2014;6(1):93-99.

21. Harrison DJ, Hughes MJ, Teitelbaum H, et al. Method of preparing emergency medicine residents for giving legal depositions. *J Am Osteopath Assoc.* 1999;99(1):28-33.

22. Miller MM, Slanetz PJ, Lourenco AP, Eisenberg RL, Kung JW. Teaching Principles of Patient-Centered Care During Radiology Residency. *Acad Radiol.* 2016;23(7):802-809.

23. Moreno MA, Kota R, McIntosh GC, Frohna JG. PEARLs of Wisdom: Impact of a New Block Conference on Pediatrics Resident Attendance, Satisfaction, and Learning. *J Grad Med Educ.* 2013;5(2):323-326.

24. Pembroke CA, Alfieri J, Biron A, Freeman C, Hijal T. Creation of an educational quality improvement program for radiation oncology residents. *Pract Radiat Oncol.* 2018;8(2):81-89.

25. Raman M, McLaughlin K, Violato C, Rostom A, Allard JP, Coderre S. Teaching in small portions dispersed over time enhances long-term knowledge retention. *Med Teach.* 2010;32(3):250-255.

26. Ramar K, Hale CW, Dankbar EC. Innovative model of delivering quality improvement education for trainees--a pilot project. *Med Educ Online.* 2015;20:28764.

27. Reznek M, Smith-Coggins R, Howard S, et al. Emergency medicine crisis resource management (EMCRM): pilot study of a simulation-based crisis management course for emergency medicine. *Acad Emerg Med.* 2003;10(4):386-389.

28. Robbins R, Sullivan S, Smith B. Implementation of an academic half day in a vascular surgery residency program improves trainee and faculty satisfaction with surgical indications conference. *Surgery.* 2018;163(6):1197-1200.

29. Schinasi DA, Kolaitis IN, Nadel FM, et al. Using Immersive Simulation to Engage Pediatric Residents in Difficult Conversations and the Disclosure of Patient Safety Events. *Cureus.* 2018;10(8):e3095.

30. Stokes W, Ruzycki S, Jainarine R, Isaac D, Cole J. The Canada-Guyana medical education partnership: using videoconferencing to supplement post-graduate medical education among internal medicine trainees. *Can Med Educ J.* 2017;8(2):e18-e24.

31. Taggar AS, Martell K, Husain S, Peacock M, Sia M, Gotto G. Exposure to radiation and medical oncology training: A survey of Canadian urology residents and fellows. *Can Urol Assoc J.* 2018;12(10):321-325.

32. Tam J, Wadhwa A. A Child With Limb Pain: A Case-Based Learning Module and Teaching Resource for Pediatric Infectious Diseases. *MedEdPORTAL.* 2017;13:10605.

33. Wagoner H, Seltz B. Attending Physicians' Perspectives of Resident Academic Half Day. *Teach Learn Med.* 2019;31(3):270-278.

34. Wong RY, Roberts JM. Real time curriculum map for internal medicine residency. *BMC Med Educ.* 2007;7:42.

35. Wong RY, Saber SS, Ma I, Roberts JM. Using television shows to teach communication skills in internal medicine residency. *BMC Med Educ.* 2009;9:9.

36. Yuasa M, Bell CL, Inaba M, et al. "You're being paged!" outcomes of a nursing home on-call role-playing and longitudinal curriculum. *J Am Geriatr Soc.* 2013;61(11):1976-1982.

37. Zastoupil L, McIntosh A, Sopfe J, et al. Positive Impact of Transition From Noon Conference to Academic Half Day in a Pediatric Residency Program. *Acad Pediatr.* 2017;17(4):436-442.

38. Zeller M, Cristancho S, Mangel J, Goldszmidt M. Back to Anatomy: Improving Landmarking Accuracy of Clinical Procedures Using a Novel Approach to Procedural Teaching. *South Med J.* 2015;108(6):310-317.
